# Supplementary figures and images for: Interstitial lung disease in a veterans affairs regional network; a retrospective cohort study
Source: PLoS One. 2021 Mar 18;16(3):e0247316. doi: 10.1371/journal.pone.0247316 (PMC7971476; doi:10.1371/journal.pone.0247316)

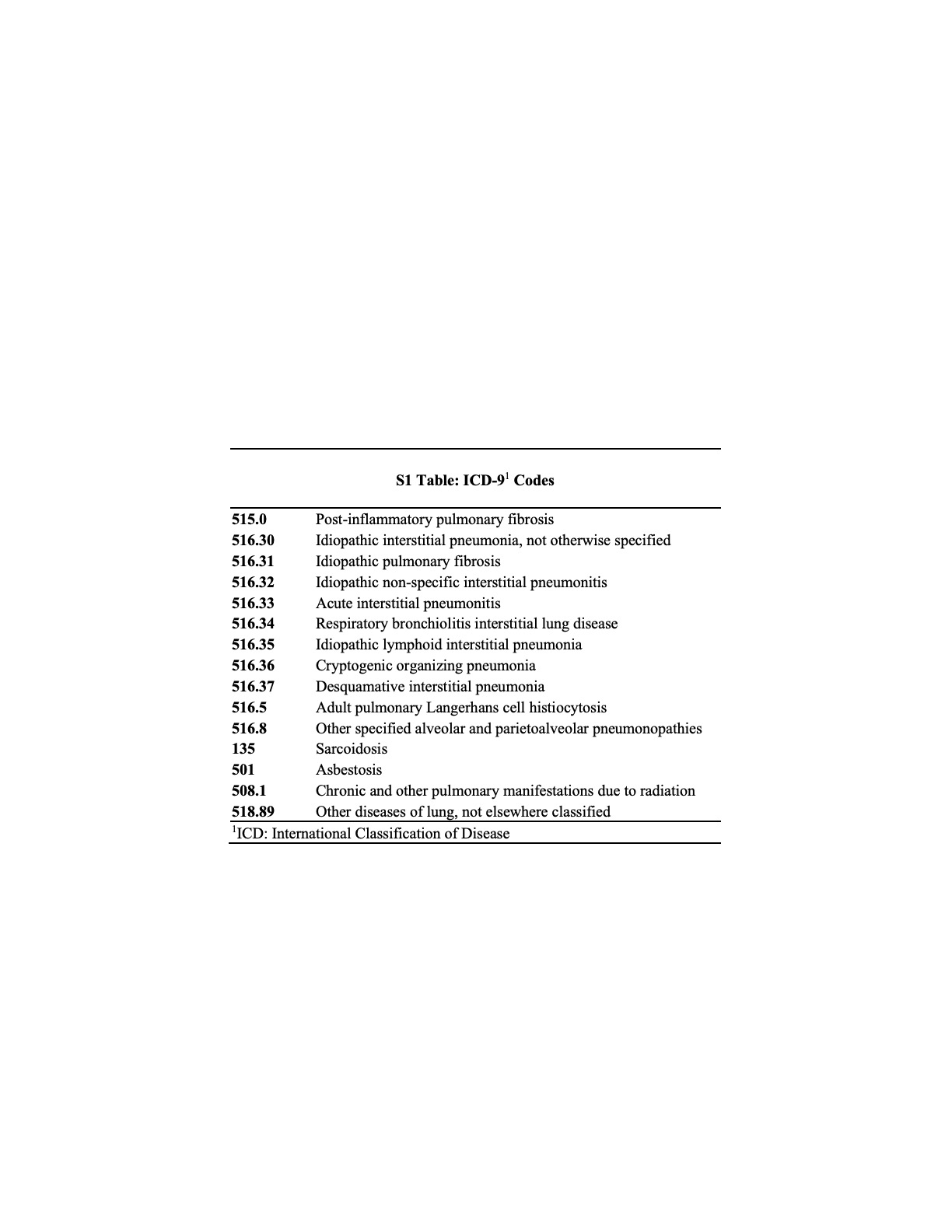

Supplement: S1 Table — (JPG) [file pone.0247316.s002.jpg]

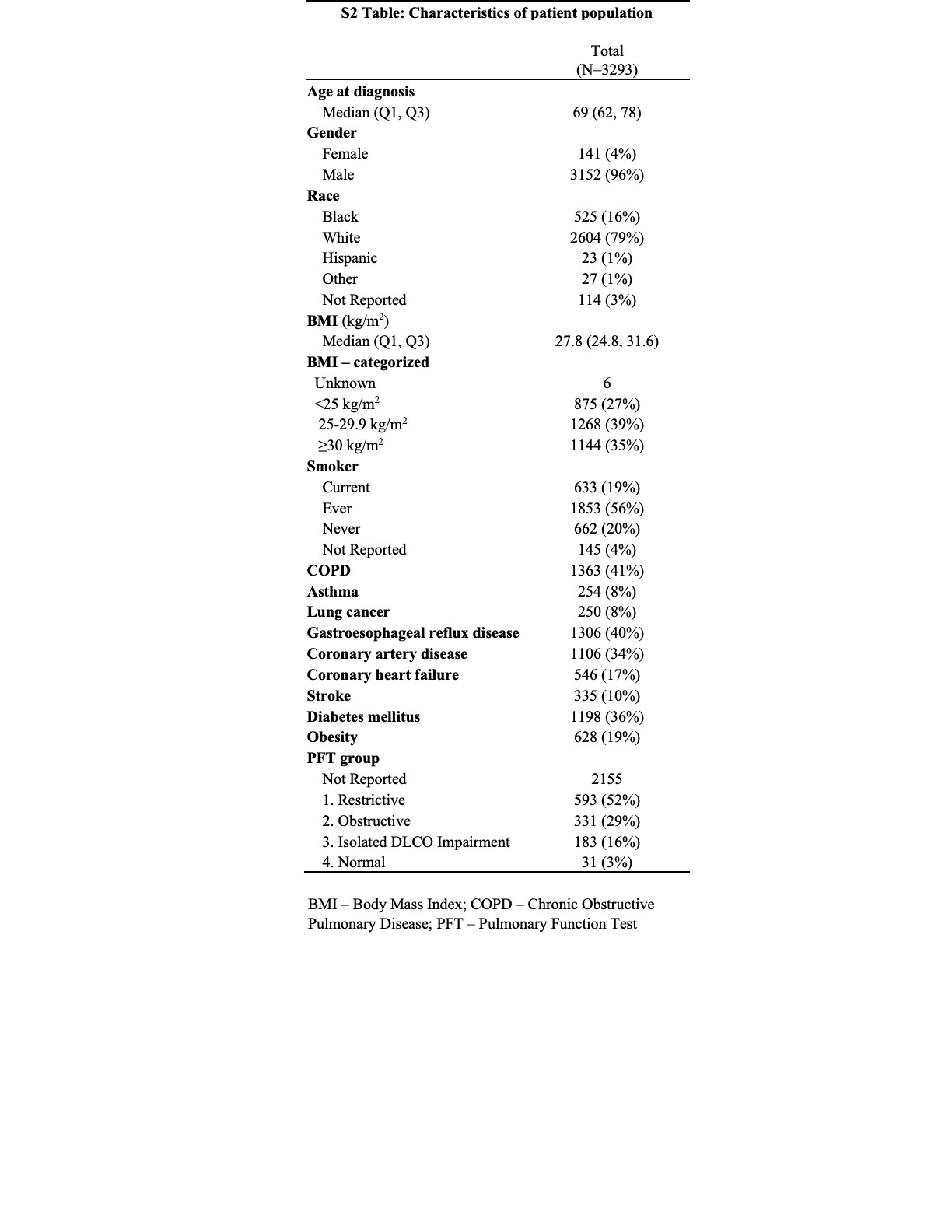

Supplement: S2 Table — (JPG) [file pone.0247316.s003.jpg]

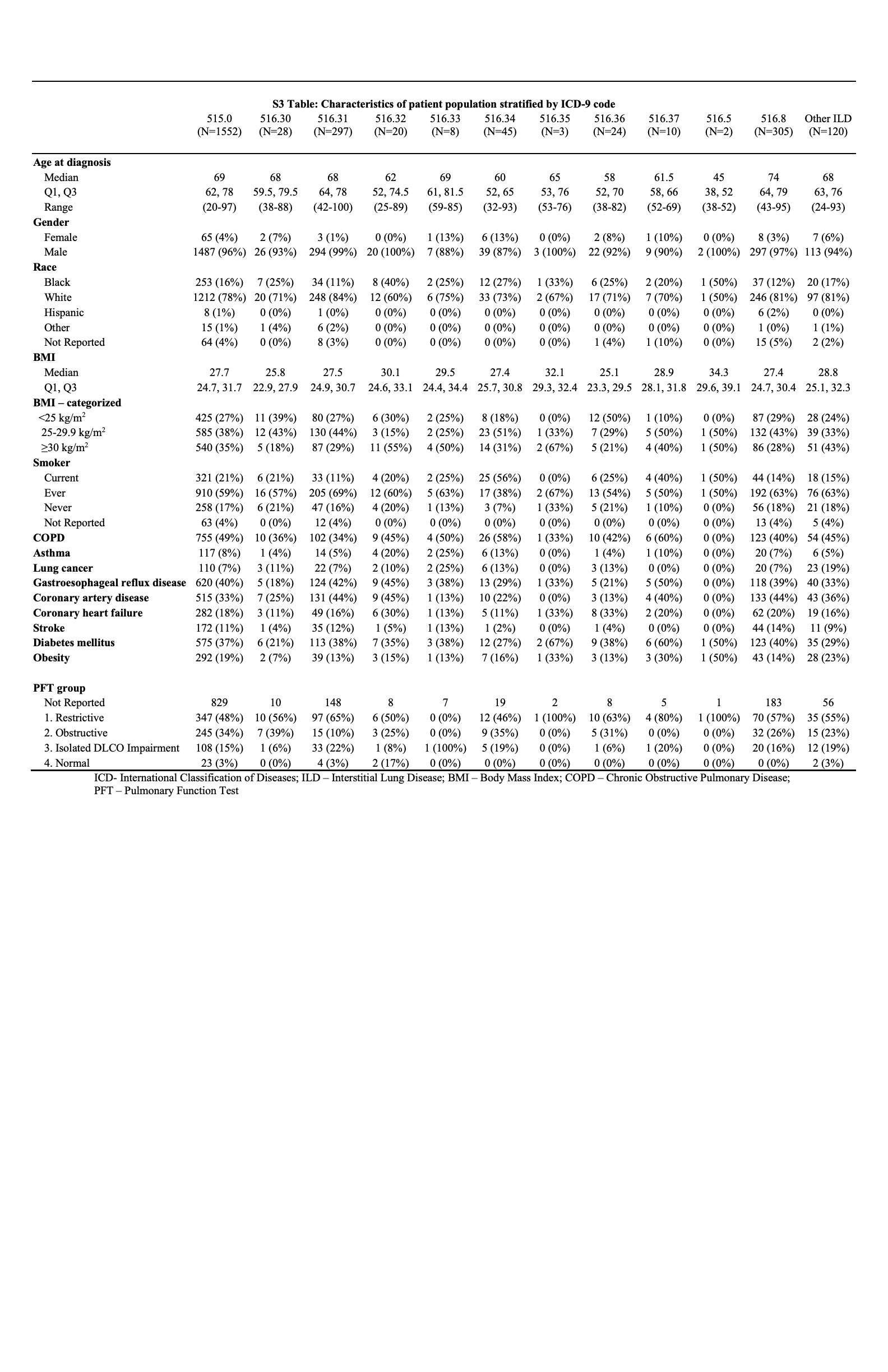

Supplement: S3 Table — (JPG) [file pone.0247316.s004.jpg]

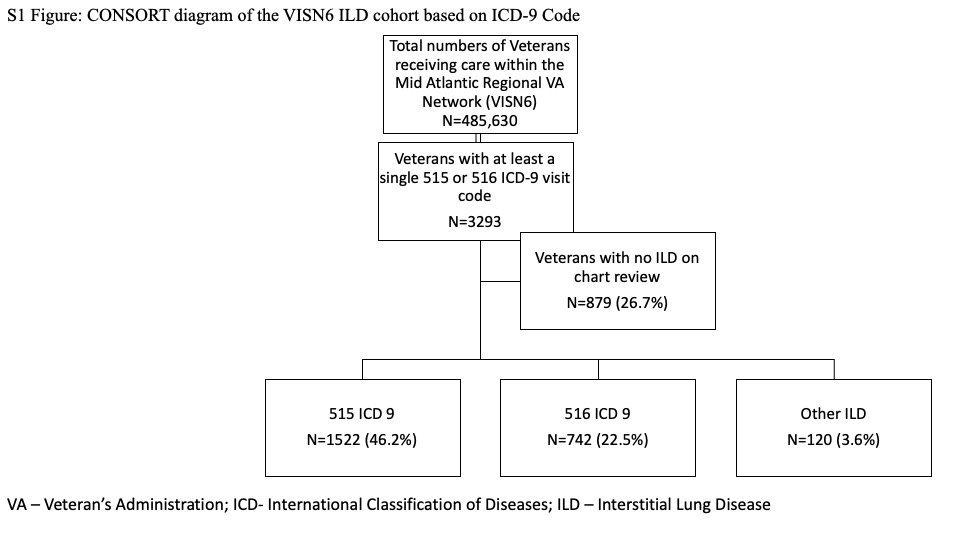

Supplement: S1 Fig — (JPG) [file pone.0247316.s005.jpg]
